# Dimorphism of [Bi_2_O_2_(OH)](NO_3_) – the ordered *Pna*2_1_ structure at 100 K

**DOI:** 10.1107/S205698902301023X

**Published:** 2023-11-30

**Authors:** Matthias Weil, Owen P. Missen, Stuart J. Mills

**Affiliations:** a Institute for Chemical Technologies and Analytics, Division of Structural Chemistry, TU Wien, Getreidemarkt 9/E164-05-1, A-1060 Vienna, Austria; b School of Earth, Atmosphere and Environment, Monash University, Clayton 3800, Victoria, Australia; c Geosciences, Museums Victoria, GPO Box 666, Melbourne 3001, Victoria, Australia; University of Aberdeen, United Kingdom

**Keywords:** crystal structure, phase transition, ordered structure, [Bi_2_O_2_]^2+^, nitrate group, group–subgroup relationship, Bärnighausen tree

## Abstract

The re-investigation of [Bi_2_O_2_(OH)](NO_3_), dioxidodibismuth(III) hydroxide nitrate, on the basis of single-crystal X-ray diffraction data revealed an apparent structural phase transition of a crystal structure determined previously (space group *Cmc*2_1_ at 173 K) to a crystal structure with lower symmetry (space group *Pna*2_1_ at 100 K). The *Cmc*2_1_ → *Pna*2_1_ group–subgroup relationship between the two crystal structures is *klassengleiche* with index 2. In contrast to the crystal structure in *Cmc*2_1_ with orientational disorder of the nitrate anion, disorder does not occur in the *Pna*2_1_ structure. Apart from the disorder of the nitrate anion, the general structural set-up in the two crystal structures is very similar: [Bi_2_O_2_]^2+^ layers extend parallel to (001) and alternate with layers of (OH)^−^ anions above and (NO_3_)^−^ anions below the cationic layer. Whereas the (OH)^−^ anion shows strong bonds to the Bi^III^ cations, the (NO_3_)^−^ anion weakly binds to the Bi^III^ cations of the cationic layer. A rather weak O—H⋯O hydrogen-bonding inter­action between the (OH)^−^ anion and the (NO_3_)^−^ anion links adjacent sheets along [001].

## Abstract 2

A structural phase transition occurs for [Bi_2_O_2_(OH)](NO_3_) between 173 K (space group *Cmc*2_1_; previous single-crystal X-ray data) and 100 K (space group *Pna*2_1_; current single-crystal X-ray data).

## 1. Chemical context

During hydro­thermal phase-formation studies of synthetic montanite, a bis­muth(III) oxidotellurate(VI) mineral with composition Bi_2_TeO_6_·*n*H_2_O (0 ≤ *n* ≤ 2/3; Missen *et al.*, 2022 ▸), small amounts of basic bis­muth(III) nitrate [Bi_2_O_2_(OH)](NO_3_) were also obtained when the starting materials Bi(NO_3_)_3_·5H_2_O (Herpin & Sudarsanan, 1965 ▸; Laza­rini, 1985 ▸), Te(OH)_6_ and KOH were reacted under hydro­thermal conditions. It is worth noting that no minerals containing both Bi and (NO_3_)^−^ have yet been found and described, with all examples of these compounds being synthetic. A routine unit-cell search at 100 K for selected crystals revealed unit-cell parameters very close to those of previously reported [Bi_2_O_2_(OH)](NO_3_) (Henry *et al.*, 2005 ▸; 173 K single-crystal X-ray data), however not with a *C*-centred but with a primitive ortho­rhom­bic unit cell. We therefore decided to determine the crystal structure based on the 100 K data and report here the results of this study.

## 2. Structural commentary

**Figure 1:**
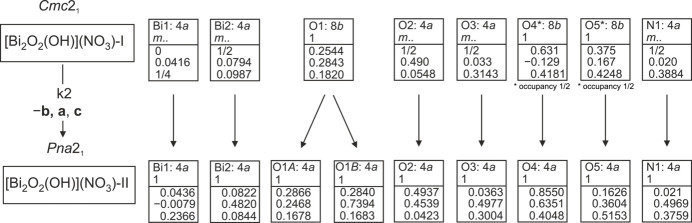
Bärnighausen tree of [Bi_2_O_2_(OH)](NO_3_), showing the detailed group–subgroup relationship between the *Cmc*2_1_ and *Pna*2_1_ structures.

The previous crystal-structure determination and refinement of [Bi_2_O_2_(OH)](NO_3_) in space group *Cmc*2_1_ resulted in a model with the nitrate anion being disordered over two possible orientations. As noted in the original report, this disorder could not be resolved: ‘Attempts to further lower the symmetry to order those anions was not successful and no supercell spots were detected on single crystal and powder diffraction data’ (Henry *et al.*, 2005 ▸). The current single-crystal X-ray diffraction data clearly revealed space group *Pna*2_1_, and the observed disorder of the nitrate anion does not prevail in the primitive unit cell, indicating that an apparent structural phase transition has taken place between 173 K and 100 K. Fig. 1 ▸ shows the Bärnighausen tree (Bärnighausen, 1980 ▸; Müller, 2013 ▸) indicating the group–subgroup relationship between the two space groups and the associated crystal structures, denoted in the following as [Bi_2_O_2_(OH)](NO_3_)-I for the 173 K data in space group *Cmc*2_1_ and as [Bi_2_O_2_(OH)](NO_3_)-II for the 100 K data in space group *Pna*2_1_. The latter is a *klassengleiche* subgroup of *Cmc*2_1_ with index 2. All atoms in [Bi_2_O_2_(OH)](NO_3_)-I that are located on sites with mirror symmetry, *viz.* atoms Bi1, Bi2, O2, O3 and N1, lie on general positions in [Bi_2_O_2_(OH)](NO_3_)-II. The O1 site in the higher-symmetry structure splits into two sites (O1*A*, O1*B*) in the lower-symmetry structure, and the two disordered (half-occupied) sites O4 and O5 fully order.

**Figure 2:**
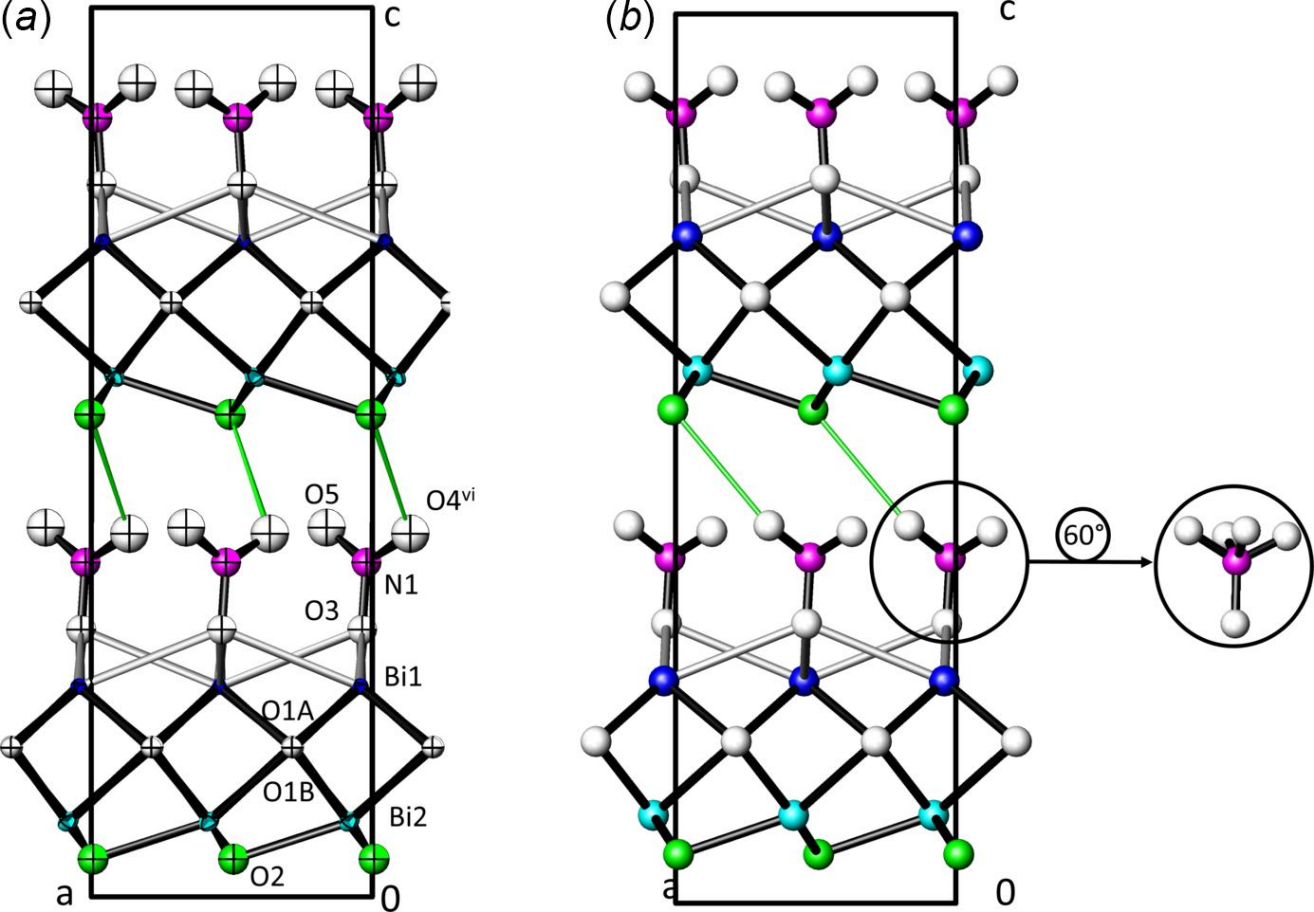
Crystal structures of the [Bi_2_O_2_(OH)(NO_3_)] polymorphs. Strong Bi—O bonds (2.20–2.62 Å) are shown as black lines, weaker Bi—O bonds (2.80–3.00 Å) as grey lines; colour code for both structures: Bi1 blue, Bi2 light blue, N1 purple, O2 associated with the OH group green, all other O atoms white; the O2⋯O hydrogen bond is displayed with green lines. (*a*) The *Pna*2_1_ structure of Bi_2_O_2_(OH)](NO_3_)-II with atoms at the 97% probability level; symmetry codes refer to Table 1 ▸ and (*b*) the *Cmc*2_1_ structure of [Bi_2_O_2_(OH)](NO_3_)-I (Henry *et al.*, 2005 ▸) with atoms shown as spheres of arbitrary radius. In the inset, the nitrate group is rotated by 60° to show the disorder present in Bi_2_O_2_(OH)](NO_3_)-I.

Apart from the ordering of the (NO_3_)^−^ group, the general structural set-up is very similar in the two crystal structures. [Bi_2_O_2_]^2+^ layers, defined by atoms Bi1, Bi2, and O1, are sandwiched between layers of (NO_3_)^−^ anions (N1, O3–O5) above and (OH)^−^ anions (O2) below. Cohesion between the resulting [Bi_2_O_2_(OH)](NO_3_) sheets is achieved through presumed weak O—H⋯O hydrogen bonds between the hydroxide anion and atom O4 of the nitrate anion (Fig. 2 ▸).

Individual bond lengths of the structure units in the two polymorphs differ slightly (Table 1 ▸); numerical values are discussed in the following paragraph only for [Bi_2_O_2_(OH)](NO_3_)-II. Within the [Bi_2_O_2_]^2+^ layer, the two Bi^III^ cations exhibit four bonds each [range 2.1964 (8)–2.657 (12) Å] to the O1*A* and O1*B* atoms that, in turn, are tetra­hedrally surrounded by the Bi^III^ cations. Such anion-centered [OBi_4_] tetra­hedra are a common structural motif in inorganic bis­muth(III) compounds (Krivovichev *et al.*, 2013 ▸). Additional strong Bi^III^—O inter­actions of 2.335 (9) and 2.493 (9) Å include the O2 atom of the hydroxide anion in the adjacent layer. On the other hand, the nitrate anion is only weakly bonded to the Bi^III^ cations of the cationic layer, with four Bi1—O3 inter­actions ranging from 2.868 (9) to 2.942 (9) Å, and another weak Bi2—O4 bond of 3.080 (10) Å. Overall, both Bi^III^ cations have eight oxygen atoms as coord­ination partners. The [Bi1O_8_] coordination polyhedron can be described as a distorted square anti­prism, whereas the [Bi2O_5_(OH)_3_] coordination polyhedron shows a significantly greater distortion (Table 1 ▸) and is difficult to derive from a simple geometric shape. In both cases, the 6*s* ^2^ free electron pair *E* of Bi^III^ located at the top of the {BiO_4_} square-pyramid (as defined by the four short Bi—O bonds) is made responsible for the distortion of the polyhedra. The resulting stereochemical effect appears to be less pronounced for the [Bi1O_8_] coordination polyhedron, but is much clearer with the [Bi2O_5_(OH)_3_] coordination polyhedron. This behaviour might possibly be explained by the stronger repulsive inter­action between *E* and the surrounding (OH)^−^ groups. The ordered (NO_3_)^−^ group in [Bi_2_O_2_(OH)](NO_3_)-II has an average N—O bond length of 1.264 Å, which is slightly longer but within the single standard deviation of the mean literature value of 1.247 (29) Å calculated for 468 N—O bonds in nitrates (Gagné & Hawthorne, 2018 ▸). The O—N—O bond angles range from 118.7 (10) to 121.3 (11)°, indicating a slight angular distortion. However, the (NO_3_)^−^ group does not deviate from planarity as observed for many nitrates, with deviations of up to 0.02 Å (Jarosch & Zemann, 1983 ▸). In [Bi_2_O_2_(OH)](NO_3_)-II, the root-mean-square deviation of fitted atoms is 0.0014 Å, with a deviation for N1 of −0.003 (10) Å from the plane defined by O3, O4(*x* − 1, *y*, *z)* and O5.

As shown in Fig. 2 ▸, the hydrogen-bonding schemes in the two [Bi_2_O_2_(OH)](NO_3_) polymorphs are different. Based on the closest O2⋯O contacts between the hydroxide and the nitrate anion, the acceptor changes from O5 [O⋯O = 2.97 (3) Å] in [Bi_2_O_2_(OH)](NO_3_)-I to O4 [O⋯O = 2.953 (14) Å] in [Bi_2_O_2_(OH)](NO_3_)-II. The closest contact of O2 to O5 in polymorph-II then is 3.017 (14) Å and that of O2 to O4 in polymorph-I is 3.16 Å. The differences in hydrogen-bonding correlate with the ordering of the (NO_3_)^−^ anion, which might be the driving force for the *Cmc*2_1_ → *Pna*2_1_ phase transition. A similar situation is found for the double salt (NH_4_)_2_SeO_4_·3NH_4_NO_3_ for which the high-temperature polymorph shows disorder of one of the nitrate groups that is fully resolved for the low-temperature polymorph (Weil *et al.*, 2023 ▸).

Bond-valence sums (Brown, 2002 ▸) were computed to validate the crystal structure model of [Bi_2_O_2_(OH)](NO_3_)-II. For the Bi^III^—O pair, the parameters of Krivovichev (2012 ▸) and for the pair N^V^—O the parameters of Brese & O’Keeffe (1991 ▸) were used (results in valence units with the numbers and types of coordination partners in parentheses): Bi1 3.02 (8, O); Bi2 2.89 (8, O); N1 4.73 (3, O); O1*A* 2.17 (4, Bi); O1*B* 2.11 (4, Bi), O2 0.84 (2, Bi); O3 2.06 (5, N + 4Bi); O4 1.72 (2, N + Bi), O5 1.63 (1, N). The results confirm the expected oxidation state of +III for Bi, and also show the underbonding of O2 as being part of the hydroxide group, and of O4 and O5 as possible acceptor atoms of hydrogen bonds.

## 3. Database survey

As described above, the crystal structures of the [Bi_2_O_2_(OH)](NO_3_) polymorphs comprise of [Bi_2_O_2_]^2+^ layers that are typical for Aurivillius phases (Henry *et al.*, 2005 ▸). [Bi_2_O_2_(OH)](NO_3_) remains the only basic bis­muth(III) nitrate for which this structural motif is known so far in the solid state. As shown for numerous other basic bis­muth(III) nitrate phases obtained under hydrolytic conditions of Bi(NO_3_)_3_·5H_2_O, the hexa­nuclear cation [Bi_6_O_4+*x*_(OH)_4–*x*_]^(6–*x*)+^ with *x* = 0 and *x* = 1 was reported to be the predominant species (Nørlund Christensen *et al.*, 2000 ▸). Later, Henry *et al.* (2005 ▸) gave a general formula of [Bi_6_O_*x*_(OH)_8-*x*_]^(10–*x*)+^ for the compositorial range of this complex cation.

A search of the Inorganic Crystal Structure Database (ICSD, version 2023_1; Zagorac *et al.*, 2019 ▸) revealed the following basic bis­muth(III) nitrate phases where this complex cation is part of the crystal structure (designation of the phases as in the original literature): [Bi_6_O_5_(OH)_3_](NO_3_)_5_·3H_2_O (Laza­rini, 1978 ▸), [Bi_6_(H_2_O)(NO_3_)O_4_(OH)_4_)](NO_3_)_5_ (Laza­rini, 1979*a* ▸), Bi_6_O_4_(HO)_4_(NO_3_)_6_·H_2_O (Sundvall, 1979 ▸), [Bi_6_O_4.5_(OH)_3.5_]_2_(NO_3_)_11_ (Henry *et al.*, 2003 ▸), [Bi_6_O_4_(OH)_4_]_0.54_[Bi_6_O_5_(OH)_3_]_0.46_(NO_3_)_5.54_ (Nørlund Christensen & Lebech, 2012 ▸), [Bi_6_O_4_(OH)_4_](NO_3_)_6_ (Henry *et al.*, 2006 ▸), [Bi_6_O_4_(OH)_4_(NO_3_)_5_(H_2_O)](NO_3_) (Miersch *et al.*, 2012 ▸), [Bi_6_O_4_(OH)_4_(NO_3_)_6_(H_2_O)_2_]·H_2_O (Miersch *et al.*, 2012 ▸), [Bi_6_O_4_(OH)_4_](NO_3_)_6_·4H_2_O (Laza­rini, 1979*b* ▸), [Bi_12_(μ_3_-OH)_4_(μ_2_-OH)_2_(μ_3_-O)_8_(μ_4_-O)_2_(NO_3_)_6_](NO_3_)_4_(H_2_O)_6_ (Liu *et al.*, 2007 ▸).

## 4. Synthesis and crystallization

Crystals of [Bi_2_O_2_(OH)](NO_3_) were obtained in a hydro­thermal reaction as a byproduct from a mixture of Bi(NO_3_)_3_·5H_2_O (0.0786 g), Te(OH)_6_ (0.0124 g) and KOH (0.0060 g) in a 3:1:2 molar ratio. The reactants were inter­mixed and 3.62 g of water was added to achieve a 2/3 inner volume of the Teflon container. The reaction vessel was enclosed in a steel autoclave, heated to 473 K and reacted for a period of 69 days under autogenous pressure. The mixture was then cooled to room temperature by removing the autoclave from the oven. The solid material obtained after the reaction time was filtered off through a glass frit, washed with mother liquor, water and ethanol and dried in air. Aside from few light-yellow crystals of [Bi_2_O_2_(OH)](NO_3_) with a plate-like form, all other products were cryptocrystalline.

## 5. Refinement

Crystal data, data collection and structure refinement details are summarized in Table 2 ▸. Inspection of the diffraction data revealed twinning by a 180° rotation about the *c* axis and inversion, which means that the Flack parameter could not be determined. After crystal structure solution, the atomic coordinates and atom labelling were adapted to the *Cmc*2_1_ structure (Henry *et al.*, 2005 ▸) for better comparison. The Bi atoms were refined with anisotropic displacement parameters, all other atoms with isotropic displacement parameters each; the H atom of the hydroxide anion (O2) could not be localized. The remaining maximum (3.03 e^−^ Å^−3^) and minimum (−3.58 e^−^ Å^−3^) electron-density peaks are located 1.63 and 1.47 Å away from Bi2 and Bi1, respectively.

## Supplementary Material

Crystal structure: contains datablock(s) I, global. DOI: 10.1107/S205698902301023X/hb8084sup1.cif

Structure factors: contains datablock(s) I. DOI: 10.1107/S205698902301023X/hb8084Isup2.hkl

CCDC reference: 2310821

Additional supporting information:  crystallographic information; 3D view; checkCIF report

## Figures and Tables

**Table 1:** Comparison of bond lengths (Å) in the *Pna*2_1_ and *Cmc*2_1_ structures of [Bi_2_O_2_(OH)](NO_3_) O atoms marked with an asterisk show half-occupancy.

[Bi_2_O_2_(OH)](NO_3_)-II (*Pna*2_1_)		[Bi_2_O_2_(OH)](NO_3_)-I (*Cmc*2_1_)	
Bi1—O1*B* ^i ^	2.203 (8)	Bi1—O1	2.226 (8)
Bi1—O1*B* ^ii^	2.207 (8)	Bi1—O1*^*a*^*	2.226 (8)
Bi1—O1*A*	2.226 (8)	Bi1—O1*^*b*^*	2.244 (8)
Bi1—O1*A* ^i^	2.292 (8)	Bi1—O1*^*c*^*	2.244 (8)
Bi1—O3^iii^	2.868 (9)	Bi1—O3*^*d*^*	2.873 (11)
Bi1—O3^ii^	2.868 (8)	Bi1—O3	2.911 (4)
Bi1—O3	2.924 (8)	Bi1—O3*^*e*^*	2.911 (4)
Bi1—O3^i^	2.941 (9)	Bi1—O3*^*f*^*	2.957 (11)
Bi2—O1*A*	2.197 (8)	Bi2—O1	2.239 (7)
Bi2—O1*B*	2.267 (8)	Bi2—O1*^*g*^*	2.239 (7)
Bi2—O2	2.334 (9)	Bi2—O2	2.341 (17)
Bi2—O1*A* ^i^	2.462 (8)	Bi2—O1*^*h*^*	2.540 (8)
Bi2—O2^i^	2.493 (9)	Bi2—O1*^*c*^*	2.540 (8)
Bi2—O1*B* ^iv^	2.619 (8)	Bi2—O2*^*h*^*	2.839 (6)
Bi2—O4^v^	3.080 (10)	Bi2—O2*^*b*^*	2.839 (6)
Bi2—O2^iv^	3.149 (9)		
N1—O5	1.251 (14)	N1—O5*	1.21 (3)
		N1—O5*^*j*^* *	1.21 (3)*^*j*^*
N1—O4^vi^	1.251 (13)	N1—O4*	1.19 (2)
		N1—O4*^*j*^* *	1.19 (2)
N1—O3	1.291 (14)	N1—O3	1.272 (16)
O2⋯O4^vii^	2.953 (14)	O2⋯O5*	2.97 (3)
		O2⋯O5^j^*	2.97 (3)

Symmetry codes for the *Pna*2_1_ structure: (i) *x* − 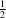 , −*y* + 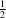 , *z*; (ii) *x*, *y* − 1, *z*; (iii) *x* + 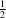 , −*y* + 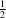 , *z*; (iv) *x* − 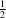 , −*y* + 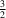 , *z*; (v) −*x* + 1, −*y* + 1, *z* − 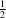 ; (vi) *x* − 1, *y*, *z*; (vii) −*x* + 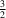 , *y* − 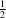 , *z* − 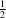 . Symmetry codes for the *Cmc*2_1_ structure: (*a*) −*x*, *y*, *z*; (*b*) − 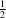 + *x*, − 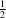 + *y*, *z*; (*c*) 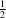 − *x*, − 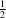 + *y*, *z*; (*d*) − 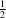 + *x*, 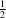 + *y*, *z*; (*e*) −1 + *x*, *y*, *z*; (*f*) − 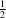 + *x*, − 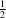 + *y*, *z*; (*g*) − 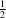 + *x*, − 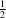 + *y*, *z*; (*h*) 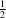 + *x*, − 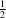 + *y*, *z*; (*j*) 1 − *x*, *y*, *z*.

**Table 2:** Experimental details

Crystal data	
Chemical formula	[Bi_2_O_2_(OH)](NO_3_)
*M*_r_	528.98
Crystal system, space group	Orthorhombic, *P* *n* *a*2_1_
Temperature (K)	100
*a*, *b*, *c* (Å)	5.3854 (13), 5.3676 (13), 17.051 (4)
*V* (Å^3^)	492.9 (2)
*Z*	4
Radiation type	Mo *K*α
μ (mm^−1^)	71.27
Crystal size (mm)	0.09 × 0.08 × 0.01

Data collection	
Diffractometer	Bruker APEXII CCD
Absorption correction	Numerical (*HABITUS*; Herrendorf, 1997 ▸)
*T*_min_, *T*_max_	0.017, 0.524
No. of measured, independent and observed [*I* > 2σ(*I*)] reflections	9853, 2798, 2407
*R*_int_	0.062
(sin θ/λ)_max_ (Å^−1^)	0.881

Refinement	
*R*[*F* ^2^ > 2σ(*F* ^2^)], *wR*(*F* ^2^), *S*	0.030, 0.057, 0.98
No. of reflections	2798
No. of parameters	48
No. of restraints	1
H-atom treatment	H-atom parameters not defined
Δρ_max_, Δρ_min_ (e Å^−3^)	3.13, −3.61
Absolute structure	Twinning involves inversion, so Flack parameter cannot be determined

Computer programs: *APEX3* and *SAINT* (Bruker, 2016 ▸), SHELXT (Sheldrick, 2015*a* ▸), *SHELXL2018/3* (Sheldrick, 2015*b* ▸), *ATOMS* (Dowty, 2005 ▸) and *publCIF* (Westrip, 2010 ▸).

## supplementary crystallographic information

### Crystal data

**Table:**

[Bi_2_O_2_(OH)](NO_3_)	*D*_x_ = 7.129 Mg m^−^^3^
*M**_r_* = 528.98	Mo *K*α radiation, λ = 0.71073 Å
Orthorhombic, *P**n**a*2_1_	Cell parameters from 3403 reflections
*a* = 5.3854 (13) Å	θ = 2.9–36.3°
*b* = 5.3676 (13) Å	µ = 71.27 mm^−^^1^
*c* = 17.051 (4) Å	*T* = 100 K
*V* = 492.9 (2) Å^3^	Plate, light yellow
*Z* = 4	0.09 × 0.08 × 0.01 mm
*F*(000) = 888	

### Data collection

**Table:**

Bruker APEXII CCD diffractometer	2407 reflections with *I* > 2σ(*I*)
ω–scans	*R*_int_ = 0.062
Absorption correction: numerical (HABITUS; Herrendorf, 1997)	θ_max_ = 38.8°, θ_min_ = 1.2°
*T*_min_ = 0.017, *T*_max_ = 0.524	*h* = −8→9
9853 measured reflections	*k* = −9→8
2798 independent reflections	*l* = −29→29

### Refinement

**Table:**

Refinement on *F*^2^	1 restraint
Least-squares matrix: full	H-atom parameters not defined
*R*[*F*^2^ > 2σ(*F*^2^)] = 0.030	*w* = 1/[σ^2^(*F*_o_^2^) + (0.0213*P*)^2^] where *P* = (*F*_o_^2^ + 2*F*_c_^2^)/3
*wR*(*F*^2^) = 0.057	(Δ/σ)_max_ < 0.001
*S* = 0.98	Δρ_max_ = 3.13 e Å^−^^3^
2798 reflections	Δρ_min_ = −3.61 e Å^−^^3^
48 parameters	Absolute structure: Twinning involves inversion, so Flack parameter cannot be determined

### Special details

**Table:**

Geometry. All esds (except the esd in the dihedral angle between two l.s. planes) are estimated using the full covariance matrix. The cell esds are taken into account individually in the estimation of esds in distances, angles and torsion angles; correlations between esds in cell parameters are only used when they are defined by crystal symmetry. An approximate (isotropic) treatment of cell esds is used for estimating esds involving l.s. planes.
Refinement. Refined as a 2-component inversion twin.

### Fractional atomic coordinates and isotropic or equivalent isotropic displacement parameters (Å^2^)

**Table:**

	*x*	*y*	*z*	*U*_iso_*/*U*_eq_	
Bi1	0.04362 (7)	−0.00785 (7)	0.23658 (3)	0.00299 (7)	
Bi2	0.08217 (8)	0.48199 (6)	0.08442 (4)	0.00432 (8)	
O1A	0.2866 (15)	0.2468 (15)	0.1678 (5)	0.0041 (15)*	
O1B	0.2840 (15)	0.7394 (15)	0.1683 (5)	0.0053 (15)*	
O2	0.4937 (17)	0.4539 (16)	0.0423 (6)	0.0097 (15)*	
N1	0.021 (2)	0.4969 (18)	0.3759 (6)	0.0091 (19)*	
O3	0.0363 (16)	0.4977 (15)	0.3004 (5)	0.0089 (14)*	
O4	0.8650 (17)	0.6351 (17)	0.4084 (6)	0.0148 (19)*	
O5	0.1626 (17)	0.3604 (16)	0.4153 (6)	0.0145 (18)*	

### Atomic displacement parameters (Å^2^)

**Table:**

	*U*^11^	*U*^22^	*U*^33^	*U*^12^	*U*^13^	*U*^23^
Bi1	0.00314 (12)	0.00303 (15)	0.00279 (14)	−0.00018 (11)	0.00001 (12)	0.0001 (2)
Bi2	0.00461 (13)	0.00439 (16)	0.00395 (15)	−0.00002 (11)	−0.00086 (14)	0.0010 (3)

### Geometric parameters (Å, º)

**Table:**

Bi1—O1B^i^	2.203 (8)	Bi2—O1B	2.267 (8)
Bi1—O1B^ii^	2.207 (8)	Bi2—O2	2.334 (9)
Bi1—O1A	2.226 (8)	Bi2—O1A^i^	2.462 (8)
Bi1—O1A^i^	2.292 (8)	Bi2—O2^i^	2.493 (9)
Bi1—O3^iii^	2.868 (9)	Bi2—O1B^iv^	2.619 (8)
Bi1—O3^ii^	2.868 (8)	N1—O5	1.251 (14)
Bi1—O3	2.924 (8)	N1—O4^v^	1.251 (13)
Bi1—O3^i^	2.941 (9)	N1—O3	1.291 (14)
Bi2—O1A	2.197 (8)		
			
O1B^i^—Bi1—O1B^ii^	75.34 (18)	O1A^i^—Bi2—O1B^iv^	64.7 (3)
O1B^i^—Bi1—O1A	116.2 (3)	O2^i^—Bi2—O1B^iv^	125.2 (3)
O1B^ii^—Bi1—O1A	75.8 (3)	Bi2—O1A—Bi1	113.5 (3)
O1B^i^—Bi1—O1A^i^	72.0 (3)	Bi2—O1A—Bi1^iii^	106.4 (3)
O1B^ii^—Bi1—O1A^i^	117.4 (3)	Bi1—O1A—Bi1^iii^	117.4 (4)
O1A—Bi1—O1A^i^	73.15 (16)	Bi2—O1A—Bi2^iii^	103.7 (3)
O1A—Bi2—O1B	72.6 (3)	Bi1—O1A—Bi2^iii^	112.2 (3)
O1A—Bi2—O2	71.7 (3)	Bi1^iii^—O1A—Bi2^iii^	102.1 (3)
O1B—Bi2—O2	77.2 (3)	Bi1^iii^—O1B—Bi1^vi^	116.1 (4)
O1A—Bi2—O1A^i^	70.34 (18)	Bi1^iii^—O1B—Bi2	107.1 (3)
O1B—Bi2—O1A^i^	104.4 (3)	Bi1^vi^—O1B—Bi2	115.2 (4)
O2—Bi2—O1A^i^	139.4 (3)	Bi1^iii^—O1B—Bi2^vii^	102.8 (3)
O1A—Bi2—O2^i^	75.1 (3)	Bi1^vi^—O1B—Bi2^vii^	107.3 (3)
O1B—Bi2—O2^i^	147.7 (3)	Bi2—O1B—Bi2^vii^	107.3 (3)
O2—Bi2—O2^i^	91.8 (3)	Bi2—O2—Bi2^iii^	98.8 (3)
O1A^i^—Bi2—O2^i^	64.8 (3)	O5—N1—O4^v^	121.3 (11)
O1A—Bi2—O1B^iv^	106.4 (3)	O5—N1—O3	120.0 (10)
O1B—Bi2—O1B^iv^	66.50 (16)	O4^v^—N1—O3	118.7 (10)
O2—Bi2—O1B^iv^	141.9 (3)		

Symmetry codes: (i) *x*−1/2, −*y*+1/2, *z*; (ii) *x*, *y*−1, *z*; (iii) *x*+1/2, −*y*+1/2, *z*; (iv) *x*−1/2, −*y*+3/2, *z*; (v) *x*−1, *y*, *z*; (vi) *x*, *y*+1, *z*; (vii) *x*+1/2, −*y*+3/2, *z*.
